# ^18^F‐Fluoro‐2‐Deoxyglucose Positron Emission Tomography/Computed Tomography Measures of Spatial Heterogeneity for Predicting Platinum Resistance of High‐Grade Serous Ovarian Cancer

**DOI:** 10.1002/cam4.70287

**Published:** 2024-10-22

**Authors:** Xin Zhang, Yuhe Lin, Dianning He, Mingli Sun, Lanlan Xu, Zhihui Chang, Zhaoyu Liu, Beibei Li

**Affiliations:** ^1^ Department of General Surgery Shengjing Hospital of China Medical University Shenyang Liaoning People's Republic of China; ^2^ Department of Oncology Shengjing Hospital of China Medical University Shenyang Liaoning People's Republic of China; ^3^ School of Health Management China Medical University Shenyang Liaoning People's Republic of China; ^4^ Department of Obstetrics and Gynecology Shengjing Hospital of China Medical University Shenyang Liaoning People's Republic of China; ^5^ Department of Radiology Shengjing Hospital of China Medical University Shenyang Liaoning People's Republic of China

**Keywords:** ovarian cancer, platinum resistance, positron emission tomography, spatial heterogeneity

## Abstract

**Background:**

The purpose of this study is to construct models for predicting platinum resistance in high‐grade serous ovarian cancer (HGSOC) derived from quantitative spatial heterogeneity indicators obtained from ^18^F‐FDG PET/CT images.

**Methods:**

A retrospective study was conducted on patients diagnosed with HGSOC. Quantitative indicators of spatial heterogeneity were generated using conventional features and Haralick texture features from both CT and PET images. Three groups of predictive models (conventional, heterogeneity, and integrated) were built. Each group's optimal model was the one with the highest area under curve (AUC). Postoperative immunohistochemical staining for Ki‐67 and p53 was conducted. The correlation between the heterogeneity indicators and scores for Ki‐67 and p53 was assessed by Spearman's correlation coefficient (*ρ*).

**Results:**

A total of 286 patients (54.6 ± 9.3 years) were enrolled. And 107 spatial heterogeneity indicators were extracted. The optimal models for each group were obtained using the Gradient Boosting Machine (GBM) algorithm. There was an AUC of 0.790 (95% CI: 0.696, 0.885) in the conventional model for the validation set, and an AUC of 0.904 (95% CI: 0.842, 0.966) in the heterogeneity model for the validation set. The integrated model achieved the highest predictive performance, with an AUC value of 0.928 (95% CI: 0.872, 0.984) for the validation set. Spearman's correlation showed that HU_Kurtosis had the strongest correlation with p53 scores with *ρ* = 0.718, while cluster site entropy had the strongest correlation with Ki‐67 scores with *ρ* = 0.753.

**Conclusions:**

Adding quantitative spatial heterogeneity indicators derived from PET/CT images can improve the prediction of platinum resistance in patients with HGSOC. Spatial heterogeneity indicators were related to Ki‐67 and p53 scores.

## 1 Background

High‐grade serous ovarian cancer (HGSOC) stands as the predominant form of epithelial ovarian cancer [1], characterized by its pronounced invasiveness and status as the primary contributor to cancer‐related mortality within the realm of gynecological cancers on a global scale [2, 3]. Of particular concern is the issue of platinum resistance [4, 5], a formidable obstacle in the treatment of ovarian cancer, which precipitates heightened rates of recurrence and diminished long‐term survival outcomes for afflicted patients [6]. Determining whether a patient has platinum resistance before treatment can help doctors judge prognosis and develop the most effective treatment strategy [7].

Platinum resistance is a complex process [8, 9, 10]. Although multiple possible mechanisms have been studied [11, 12], the results have led to little translational correlation in the clinical environment [13]. Currently, there is limited information on reliable non‐invasive predictive factors related to chemotherapy resistance in ovarian cancer. Research has shown that tumor heterogeneity may be one of the reasons for inconsistent responses to the same treatment regimen [14]. This heterogeneity includes not only intra‐tumor heterogeneity but also inter‐tumor heterogeneity, the latter of which pertains to the variation between the primary tumor and metastatic lesions, or between different metastatic lesions, also known as spatial heterogeneity.

Image‐based radiomics can effectively evaluate the intra‐tumor and spatial heterogeneity of ovarian cancer. The use of radiomic features can predict the residual disease [15] and chemotherapy response of ovarian cancer [16, 17, 18]. Veeraraghavan et al. developed measures for tumor heterogeneity based on computed tomography (CT), which can better predict platinum resistance [19] in HGSOC. ^18^F‐fluoro‐2‐deoxyglucose (^18^F‐FDG) positron emission tomography (PET) CT, commonly known as PET/CT, is a valuable examination for evaluating the degree of malignancy of ovarian cancer and areas involved throughout the whole body. Compared with simple CT, it can better locate metastatic lesions [20] and has superior specificity for the detection of metastasis [21]. Therefore, we hypothesized that quantitative heterogeneity indicators based on ^18^F‐FDG PET/CT will better predict platinum resistance.

Contrastingly, Ki‐67 and p53 are immunohistochemical markers commonly used in clinical practice. In ovarian cancer, Ki‐67 expression has been linked to poor prognosis and the proliferation capacity of tumor cells [22, 23, 24]. Compared to patients with normal p53 expression, patients with p53 overexpression have a shorter progression time and are more prone to platinum resistance [25, 26].

The aim of this research was to construct spatial heterogeneity indicators through the analysis of ^18^F‐FDG PET/CT images, then to investigate the potential enhancement of predictive accuracy for platinum resistance in HGSOC by incorporating these indicators. Furthermore, we evaluated the correlation between spatial heterogeneity indicators and the levels of immunohistochemical markers.

## 2 Methods

### 2.1 Participants and Procedures

Data were collected from records of patients with pathologically confirmed primary HGSOC in our two affiliated hospitals between 3 January 2010 and 28 December 2020. To be eligible, patients had to undergo cytoreductive surgery following ^18^F‐FDG PET/CT scan, the surgery had to take place at our facilities, and patients had to be pathologically diagnosed with HGSOC. Criteria for exclusion included failure to identify primary ovarian lesions on PET or CT images, presence of significant artifacts in CT images hindering tumor observation, inability to detect metastasis or absence of metastatic lesions, and incomplete data. Platinum‐based chemotherapy was administered to all the patients after surgery. Platinum resistance was characterized by a period of < 6 months without platinum treatment following the initial therapy [27].

### 2.2 Image Acquisition

All participants underwent PET/CT imaging utilizing the Discovery PET/CT 690 system (GE Healthcare, USA) following established guidelines [28, 29]. The ^18^F‐FDG utilized in the scans was manufactured by the hospital, boasting a purity exceeding 99% and a pH range of 4.5–8.5. Prior to the PET/CT examination, participants were required to fast for a minimum of 6 h and have their blood glucose levels assessed. Blood glucose levels were required to be below 7 mmol/L, and the injection of ^18^F‐FDG (3.7 MBq/kg) was administered through the brachial vein. PET scans should be conducted at a rate of 1.5 min per bed, with a total of 8–9 beds acquired, encompassing the anatomical range from the apex of the skull to the mid‐femur. Spiral CT scanning should utilize a layer thickness of 3.8 mm, a tube voltage of 120 kV, and an automatic tube current ranging from 15 to 180 mA. Reconstruction of attenuation‐corrected PET images should employ the ordered subset expectation maximization (OSEM) algorithm with 24 subsets and two iterations, in addition to utilizing time‐of‐flight and point spread function techniques. In terms of applied activity, ^18^F‐FDG had a median of 314.55 ± 62.69 MBq.

### 2.3 Image Segmentation

The CT and PET image sequences were imported into the IntelliSpace Discovery platform version 3.0 software developed by Philips Healthcare in Best. This software has the capability to simultaneously present the corresponding layers of PET and CT images through the utilization of the “Research Oncology Suite” plug‐in unit, as seen in Appendix S1.1. Utilizing the metabolic information provided by PET images and the information provided by CT images, researchers defined the high FDG uptake region of the lesion as the volume of interest (VOI). This approach is predicated on the observation that the majority of HGSOC lesions exhibit a cystic‐solid composition. Typically, the cystic component contains a minimal number of tumor cells, whereas the solid component harbors a substantial number of tumor cells, which demonstrate elevated metabolic activity on PET images. Using the solid component of the lesion to generate indicators of spatial heterogeneity may more accurately capture the discrepancies among tumor cells.

The identification of peritoneal metastatic lesions in HGSOC was based on the anatomical abdominopelvic 9‐region method [30, 31]. By intersecting two sagittal planes and two transverse planes, the abdomen is anatomically divided into nine regions. The two sagittal planes split the abdominal cavity into three equal segments. The two transverse planes are the lowest margin of costal arch and the plane of anterior superior iliac spine. The umbilical region is designated area 0 (AR0). Starting from the right upper part to the right middle part, the regions are numbered AR1‐AR8 clockwise [32], as seen in Appendix S1.2. The nine regions were sequentially assessed for each patient. If a lesion was identified in a particular region, the VOI was delineated accordingly. In other words, each patient had 2–10 VOIs (including primary lesion). Each VOI was required to be greater than 5 × 5 × 5 mm. We involved two professional researchers (SBH and ZDG, who have interpreted PET/CT images for 12 and 15 years, respectively) to evaluate all the regions, and disagreements were resolved by a senior researcher (GZY, with 23 years of experience). SBH outlined the VOIs of all lesions. To assess the intra‐ and inter‐group consistency, SBH and ZDG repeated the segmentation process independently on 30 randomly chosen cases after a lapse of 1 month.

### 2.4 Using Conventional Features to Generate Spatial Heterogeneity Indicators

Eight conventional features were recorded for each VOI in the pathological area. Four CT features included CT value, minor axis length, major axis length, and pixel number. Among the PET features were total lesion glycolysis and three standard uptake values (SUV): mean SUV, peak SUV, and maximum SUV. The following 13 statistics were calculated for each conventional measurement at the patient level: mean, standard deviation, median, quartile deviation, mode, variance, range, standard error of the sample mean, uncorrected sum of squares, corrected sum of squares, coefficient of variation, kurtosis, and skewness. Accordingly, for each patient, 13 × 8 = 104 spatial heterogeneity indicators were generated. The detailed method can be found in Appendix S1.3.

### 2.5 Using Texture Features to Generate Spatial Heterogeneity Indicators

The relevant functions in the Computational Environment for Radiological Research package were used to calculate CT texture feature parameters [33]. The radiomic texture features were defined in accordance with the Image Biomarker Standardization Initiative guidelines [34]. Similar voxels were subsequently classified into clusters based on the similarity of their textural features. Initially, the CT images underwent rescaling to utilize 256 gray levels, followed by discretization using a bin width of 32. Subsequently, the gray‐level co‐occurrence matrix was computed for each voxel within the VOI [35]. Four unrelated features, namely Energy, Entropy, Contrast, and Homogeneity, also known as Haralick texture features, were extracted at the voxel level. The Gaussian Mixture model clustering algorithm [36] was used to convert voxels into subregions with homogeneous textures. The textural dissimilarities between subregion pairs were calculated as the Euclidean distance [19] and displayed as a dissimilarity matrix. A spatial heterogeneity indicator called cluster site entropy (cSE) was calculated using the dissimilarity matrix [37]. The two‐dimensional histogram was designated the group diversity matrix (GDM), from which two spatial heterogeneity indicators, cluster diversity (cluDev) and cluster standard deviation (cludiss), were calculated [32].

### 2.6 Immunohistochemical Staining and Scoring

Following surgery, all patients underwent pathological section preparation, a process carried out by the pathology department at our hospital. The pathological sections were preserved in 10% formaldehyde and encased in paraffin. In order to ensure precise quantification of the degree of immunohistochemical staining, we scanned the immunohistochemical sections to generate digital whole slide images using a Pannoramic MIDI scanner (3DHISTECH, Hungary). Furthermore, we calculated the immunohistochemical scores using Aipathwell (Servicebio, China). This software utilizes artificial intelligence technology, specifically deep learning principles, to train algorithms using extensive data sets and incorporate them into automated image analysis processes. The outcomes were determined through various stages, including tracking, color selection, operation, and analysis [38]. The software initially identified and outlined the region of interest on the tissue automatically. Subsequently, utilizing whole slide images, it autonomously conducted positive assessments, identified the nucleus, and extended the cytoplasmic boundaries. It then computed the quantity and size of weak, moderate, and strong positive cells, in addition to various parameters including integrated optical density and tissue area. Ultimately, it systematically calculated the test region at a high level of magnification using the initial data and algorithmic formulas, culminating in the generation of an analytical report. To assess the link between immunohistochemical scores and spatial heterogeneity, Spearman's rank correlation coefficient was used.

### 2.7 Platinum Resistance Feature Selection and Modeling

Three groups of prediction models were established: conventional, heterogeneity, and integrated models. The collected data used to establish platinum resistance models is seen in Appendix S1.4. For each group of models, H_2_O Automatic Machine Learning (H2O AutoML) was used for feature selection and modeling (https://docs.h2o.ai/h2o/latest‐stable/h2o‐docs/automl.html). This method can perform multiple algorithms simultaneously, like GBM, discriminative random field (DRF), extremely randomized trees (XRT), generalize linear model (GLM), Stacked Ensemble and DeepLearning. Following the completion of automated learning, the H2O system will generate the top 10 models exhibiting the highest performance according to their respective parameters. This research sought to identify the optimal model by evaluating the area under the curve (AUC) metric. The training process adopts 5‐fold cross validation. The training set and validation set were from the Nanhu Campus (NH) and Huaxiang Campus (HX) of our hospital, respectively.

### 2.8 Statistical Analysis

The Kolmogorov–Smirnov test was utilized to assess the normality of the data. Normalized descriptive data were shown as means ± standard deviations, while non‐normalized data was shown as medians, upper quartile (1st Qu.), and lower quartile quartiles (3rd Qu). *t*‐tests were used for comparisons between groups composed of normally distributed data, Mann–Whitney *U* tests for non‐normally distributed data, and chi‐square tests for enumeration data. The intraclass correlation coefficient (ICC) [39] was used to evaluate intra‐ and inter‐group consistency.

Spatial heterogeneity indicator extraction was carried out utilizing the software MATLAB R2022a (MathWorks, USA), while platinum resistance feature selection and modeling were performed using R software (version 4.1.0). Key packages utilized in the analysis included “corrplot,” “lattice,” “rms,” “ggplot2,” and “h_2_o.” Statistical significance was determined at a *p* < 0.05 (two‐tailed). The data and analysis scripts for this study can be accessed on GitHub at https://github.com/beibei22008/platinum‐resistance_HGSOC.

## 3 Results

### 3.1 Patient Characteristics

**FIGURE 1:**
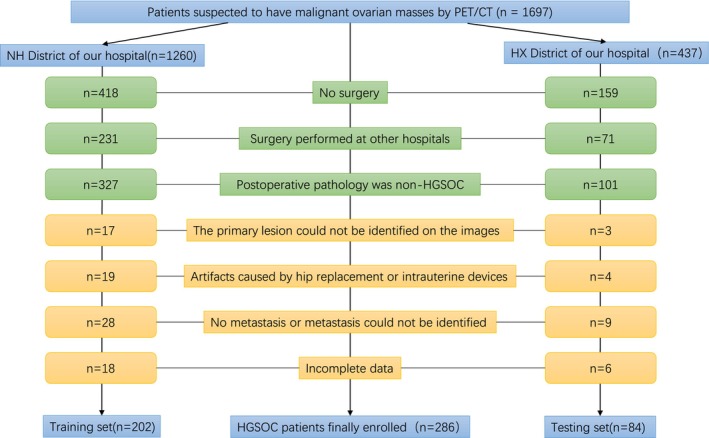
Flowchart of the enrolled patients of the training set and validation set.

Initially, 1697 patients performed PET/CT scans and were diagnosed with suspected malignant ovarian masses. After screening, 286 patients were ultimately included in this study, as shown in Figure 1. There were no significant differences (*p* > 0.05) in the clinical information and lesion characteristics between participants in the training set and those in the validation set. Tables 1 and 2 provided summaries of the clinical information and lesions' characteristics of the participants.

**TABLE 1:** Clinical characteristics and demographic of participants.

	Training set (*n* = 202)	Validation set (*n* = 84)	*p*
Age: median (1st Qu., 3rd Qu.)	54 (47, 61)	55 (48, 60)	0.53
CA125: median (1st Qu., 3rd Qu.)	867.0 (403.0, 1779.3)	867.1 (503.8, 1717.8)	0.67
FIGO stage (%)			
IIB	3 (1.49%)	1 (1.19%)	0.62
IIIA	6 (2.97%)	0 (0.00%)	
IIIB	19 (9.41%)	8 (9.52%)	
IIIC	110 (54.46%)	51 (60.71%)	
IVA	37 (18.32%)	12 (14.29%)	
IVB	27 (13.37%)	12 (14.29%)	
Ascites volume: median (1st Qu., 3rd Qu.; Unit: mL)	1000 (200, 3000)	1750 (200, 3000)	0.36
Ascites (%)			
No	10 (4.95%)	5 (5.95%)	0.92
Bloody	56 (27.72%)	24 (28.57%)	
Non‐bloody	136 (67.33%)	55 (65.48%)	
Residual tumors (%)			
No	119 (58.91%)	45 (53.57%)	0.71
≤ 1 cm	42 (20.30%)	19 (22.62%)	
> 1 cm	47 (20.79%)	20 (23.81%)	
Survival: median (1st Qu., 3rd Qu.; Unit: days)			
Progression free survival	637.50 (412.00, 877.25)	635.55 (401.75, 871.00)	0.72
Overall survival	1024.50 (738.00, 1628.50)	949.55 (632.98, 1569.43)	0.09
Platinum‐resistance	64 (31.68%)	33 (39.29%)	0.22

Abbreviation: FIGO, federation of gynecology and obstetrics.

**TABLE 2:** Lesions' characteristics of participants.

	Training set (*n* = 202)	Validation set (*n* = 84)	*p*
Number of metastatic lesions: median (1st Qu., 3rd Qu.)	6.00 (3.00, 8.00)	7.00 (4.25, 9.00)	0.21
Metastatic lesions (%)			
Left lower	130 (64.36%)	57 (67.86%)	0.59
Pelvis	170 (84.16%)	75 (89.29%)	0.35
Right lower	122 (60.40%)	55 (65.48%)	0.50
Left flank	85 (42.08%)	38 (45.24%)	0.69
Central	92 (45.54%)	40 (47.62%)	0.80
Right flank	140 (69.31%)	61 (72.62%)	0.67
Left upper	129 (63.86%)	54 (64.29%)	0.53
Epigastrium	83 (41.09%)	45 (53.57%)	0.07
Right upper	141 (69.81%)	61 (72.62%)	0.67
Invasion mode (%)			
No	28 (13.86%)	4 (4.76%)	0.06
Nodular	38 (18.81%)	21 (25.00%)	
Predominantly nodular	45 (22.28%)	12 (14.29%)	
Predominantly infiltrate	45 (22.28%)	24 (28.57%)	
Infiltrate	46 (22.77%)	23 (27.38%)	
Metastasis of lymph nodes (%)			
Pelvic	67 (33.17%)	32 (38.10%)	0.50
Middle abdominal	67 (33.17%)	30 (35.71%)	0.68
Upper abdominal	62 (30.69%)	29 (34.53%)	0.58
Distant	29 (14.36%)	13 (15.48%)	0.86

### 3.2 Prediction Models for Platinum Resistance

**FIGURE 2:**
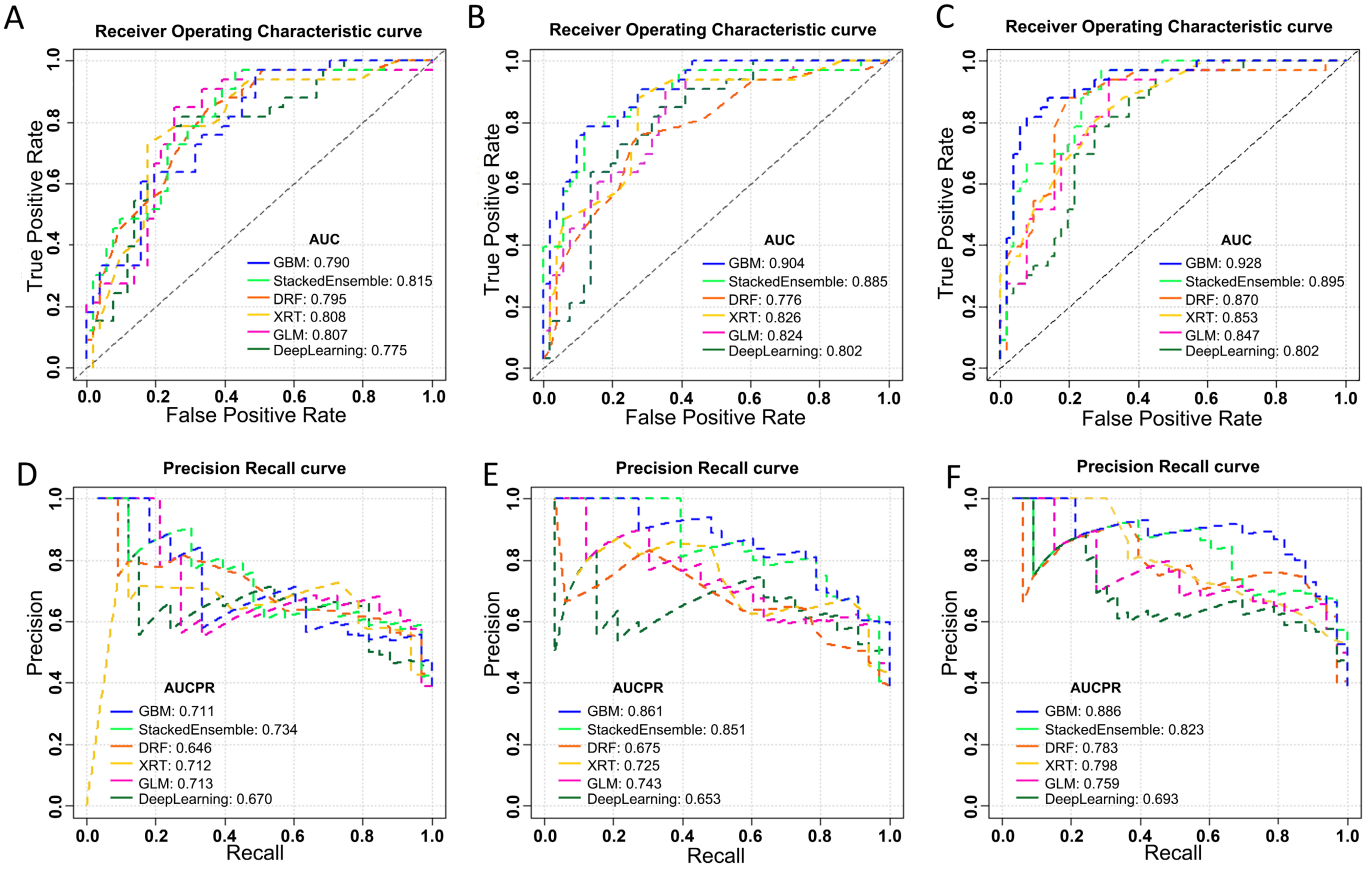
The performance comparison of models in each group. The area under the ROC curve (A–C) and area under the precision‐recall curve (D–F) of the conventional model, heterogeneity model, and integrated model, respectively. DRF, discriminative random field; GBM, gradient boosting machine; GLM, generalized linear model; XRT, extremely randomized trees.

The heterogeneity indicators showed good consistency both in intra‐ and inter‐group, with ICC > 0.75. The collected data were used to establish three groups of platinum resistance models. After H_2_O AutoML, the GBM model proved to be the optimal model for conventional, heterogeneity, and integrated models. A comparison of the AUC and area under curve of precision recall (AUCPR) for the different learning algorithms in each group models is shown in Figure 2. The AUC and AUCPR metrics of the GBM model surpassed those of the other algorithmic models. Consequently, the GBM model was selected as the optimal model within each group for subsequent comparisons.

**FIGURE 3:**
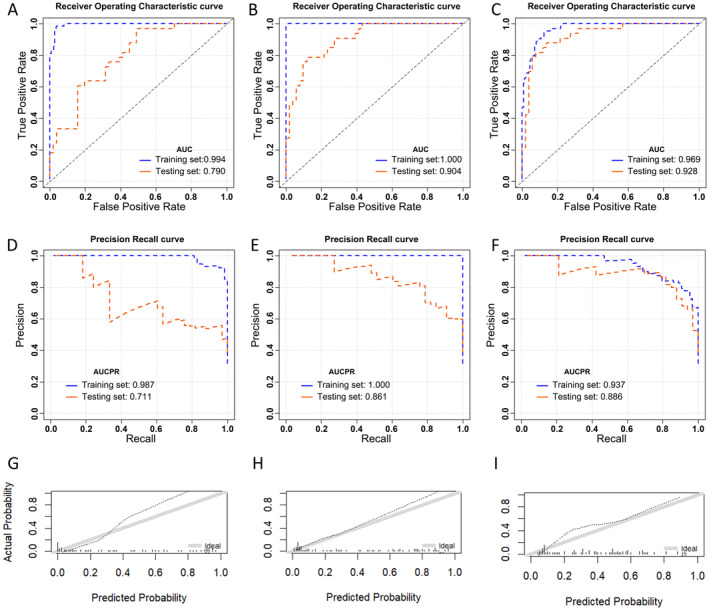
The performance of the optimal models. The area under the ROC curve (A–C), area under the precision‐recall curve (D–F), and calibration curves (G–I) in the validation set of the conventional model, heterogeneity model, and integrated model, respectively. AR8, peritoneal metastasis in right flank area; cSE, cluster site entropy; Dis_LN, distant lymph node metastasis; DRF, discriminative random field; FIGO_stage, federation of gynecology and obstetrics stage; GBM, gradient boosting machine; GLM, generalize linear model; HU‐Kurtosis, Kurtosis value of CT value; Max_SM, the standard error for the sample mean of maximum standard uptake value; Min_SM, the standard error for the sample mean of minor axis length; Num_AR, number of areas of lesions; Peak_Skewness, the skewness of peak standard uptake value; Sur_status, surgical excision status; SUVmean, mean standard uptake value; TLG‐Kurtosis, Kurtosis value of the total amount of glucose decomposition; Upper_LN, upper abdominal lymph node metastasis; Vol_SM, the standard error for the sample mean of the volume; XRT, extremely randomized trees.

We then compared the chosen optimal models in each group (conventional, heterogeneity, and integrated model). The order of the AUC in the validation set was as follows: integrated (0.928, 95% CI: 0.872, 0.984) > heterogeneity (0.904, 95% CI: 0.842, 0.966) > conventional (0.790, 95% CI: 0.696, 0.885) model. The integrated model had the best prediction performance Figure 3. showed AUC, AUCPR, and calibration curves for the selected optimal models within each group.

Focusing on the integrated model, the optimal model was GBM_grid_1_AutoML_2_20230923_170056_ Model _4. The detailed evaluation indicators of the models were summarized in Table 3. Cross‐Validation showed the mean accuracy was 0.831 ± 0.045, and the mean specificity was 0.820 ± 0.110.

**TABLE 3:** The evaluation indicators for the integrated model.

	Training set	5‐fold cross‐validation	Validation set
Mean squared error	0.0873911	0.142697	0.1228745
Root mean squared error	0.2956199	0.3777525	0.3505346
Logarithmic loss	0.3032489	0.4347732	0.3938861
Mean per‐class error	0.09035326	0.1837636	0.1301248
Area under the roc curve	0.9694293	0.8602808	0.9281046
Area under the precision‐recall curve	0.9373951	0.7535669	0.8863363
Gini coefficient	0.9388587	0.7205616	0.8562092
*R* ^2^	0.8379682	0.4588393	0.602902

**FIGURE 4:**
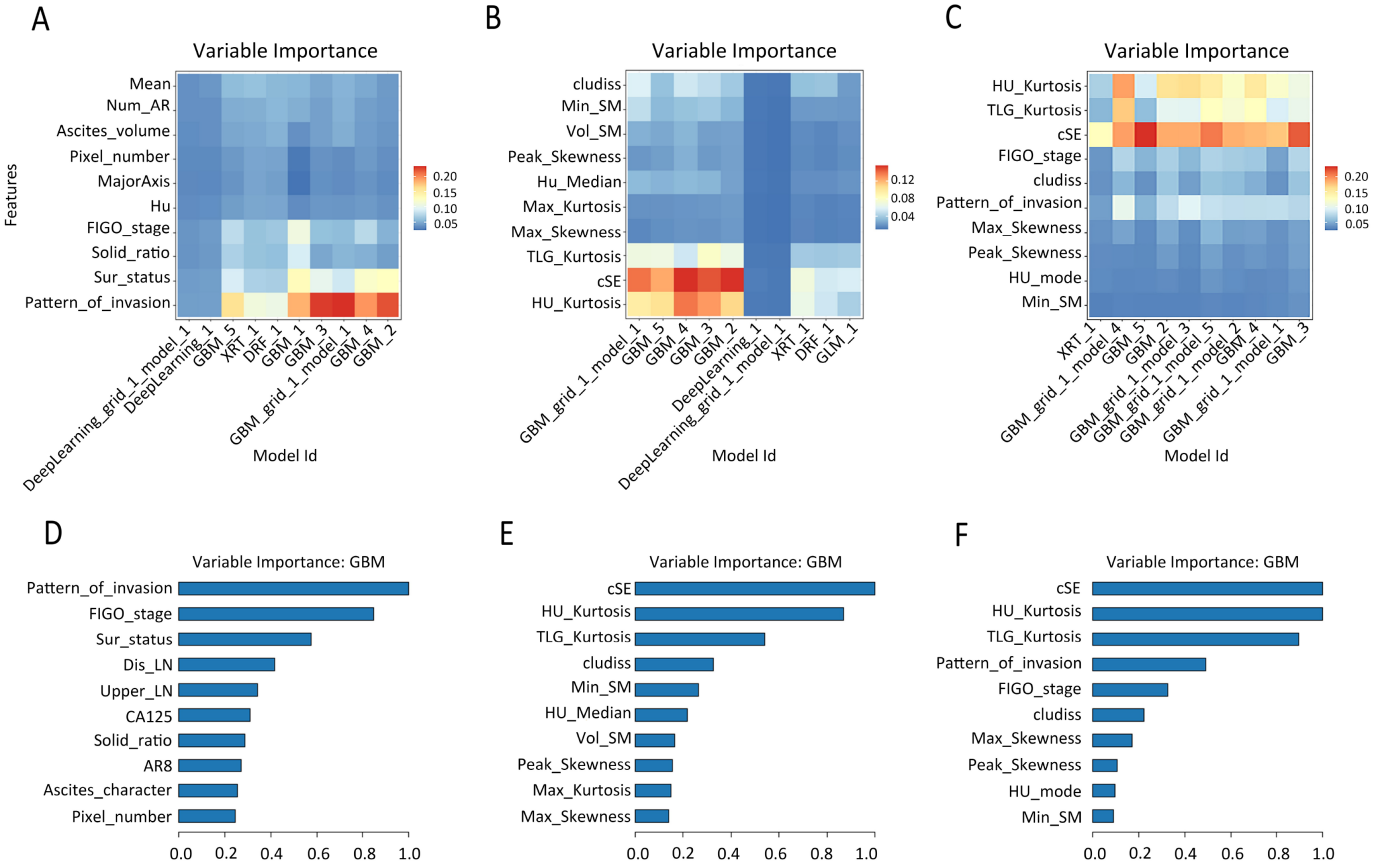
Variable analysis of models. The variable importance heatmap (A–C) and variable importance plots (D–F) of the optimal models in each group (conventional models, heterogeneity models, and integrated models), respectively.

To conduct a more detailed examination of the internal feature composition and the relative significance of each feature within the models, we employed Variable Importance Heatmaps and Variable Importance Plots. The Variable Importance Heatmap (Figure 4A–C) displayed the percentage values of the top ten most significant features across multiple models, facilitating the selection of optimal models. And the Variable Importance Plot (Figure 4D–F) illustrated the relative importance of the most critical features within each model, with each feature's importance normalized on a scale from 0 to 1.

**FIGURE 5:**
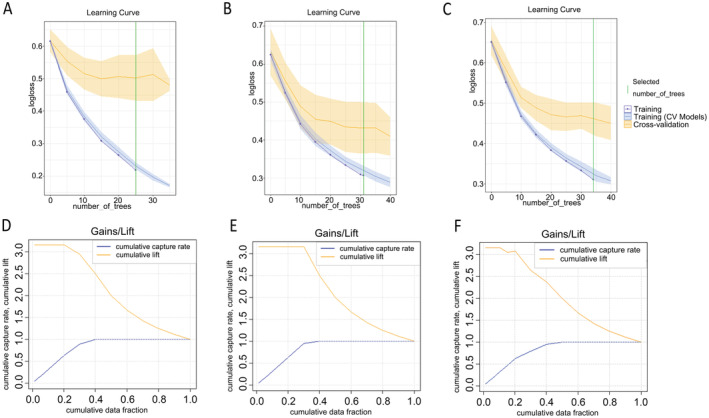
The learning curves and gains/lift graphs. The learning curves (A–C) and gains/lift graphs (D–F) of the optimal models in each group (conventional models, heterogeneity models, and integrated models), respectively.

The learning curve plot (Figure 5A–C) illustrated the error metric's dependence on the learning progress of each model. Gains/Lift graphs (Figure 5D–F) assessed the improvement and overall accuracy of the model in predicting platinum resistance.

### 3.3 Correlation Analysis

**FIGURE 6:**
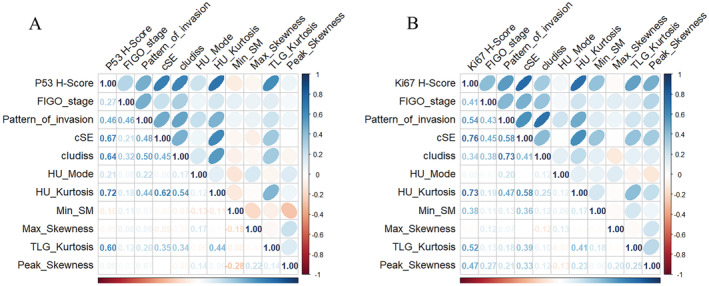
Correlation analysis. The correlation analysis graph between the selected features and immunohistochemical scores of p53 (A) and Ki‐67 (B). The number represented in the chart shows the Spearman's correlation coefficient values of the two variables. cSE, cluster site entropy; FIGO_stage, federation of gynecology and obstetrics stage; HU‐Kurtosis, Kurtosis value of CT value; Min_SM, the standard error for the sample mean of minor axis length; TLG‐Kurtosis, Kurtosis value of the total amount of glucose decomposition.

Based on the Variable Importance Plots of the integrated model, we selected the ten features with the highest scores for the next correlation analysis. Eighty‐six patients have performed the p53 immunohistochemical staining. Spearman's correlation analysis revealed a significant positive association between HU_Kurtosis and the immunohistochemical score for p53 (*ρ* = 0.718, *p* < 0.01), indicating a strong correlation. Among the 84 patients who underwent Ki‐67 immunohistochemical staining, cSE exhibited the strongest positive association between the immunohistochemical level for Ki‐67 with correlation coefficient *ρ* = 0.759 (*p* < 0.01), as illustrated in Figure 6.

## 4 Discussion

This study established spatial heterogeneity indicators through two dimensions of two modalities from PET/CT images, and the predictive model for heterogeneity that was developed demonstrated superior accuracy in predicting resistance to platinum compared to the traditional model. Additionally, this research discovered a correlation between the levels of Ki‐67 and p53 expression and markers of spatial heterogeneity.

In the process of establishing a predictive model for platinum resistance, the best model selected using Automatic Machine Learning was the GBM. As a decision‐tree‐based algorithm for machine learning, the GBM is currently considered the most advanced algorithm for predicting tabular data and has achieved excellent results in various clinical modeling competitions [40]. It has many advantages over other decision‐tree‐based algorithms, such as being able to disassemble into simple decision‐tree‐based learners, providing more interpretable programs, and allowing researchers to understand their internal structure. The GBM algorithm naturally incorporates interactions between covariates. Furthermore, a gradient‐boosting predictor can be utilized to handle missing values without requiring imputation of missing data, thus increasing the pool of patients for modeling [40]. Moreover, the training set makes use of a 5‐fold cross‐validation method in order to avoid overfitting and adjust the parameters [41]. To date, GBM has been used to predict cancer‐specific survival in resected intrahepatic cholangiocarcinoma [42], diabetes mellitus [43], outcomes of primary sclerosing cholangitis [44], survival in colorectal cancer [45], and risk in patients with gastrointestinal bleeding [46]. However, there are few applications of automatic machine learning in ovarian tumors. Some studies have confirmed that the radiomics of CT [47, 48] and MR [49] features help diagnose malignant and benign ovarian cancers. A recent publication utilized 3D CT radiomics to differentiate between Type I and Type II epithelial ovarian cancers through the application of various machine learning classifiers prior to surgical intervention [50]. It is foreseeable that machine learning will play an important future role in many medical fields.

cSE and other spatial heterogeneity indicators are important components of the platinum‐resistant model. Veeraraghavan et al. [19] found that there were more primary platinum‐resistant patients in a group of patients with high heterogeneity on CT, and the classifier using the heterogeneity indicator of CT imaging and clinical genomics had the highest sensitivity in predicting platinum resistance. The results of our study support this finding. Heterogeneity within the ovarian lesion and between ovarian lesions is inherent. CT‐based spatial heterogeneity indicators have been confirmed to be related to the enrichment of the WNT pathway and stromal cell types [19]. Furthermore, WNT/β‐enrichment of catenin signaling is related to immune rejection [51]. A prior study [35] discovered that amplification of 19q12, specifically involving CCNE1, predominantly manifests in individuals exhibiting high texture heterogeneity. Furthermore, this amplification of CCNE1 is linked to unfavorable prognosis and resistance to chemotherapy in patients with HGSOC [52]. Heterogeneity CT features are related to the BRCA mutation status in HGSOC patients [53, 54] and the abundance of multiple proteins in vivo [55].

We confirmed that spatial heterogeneity indicators may be associated with the immunohistochemical scores of Ki‐67 and p53. Ki‐67 is widely expressed in proliferating cells at tumor sites, and has been established as a marker correlated with tumor invasiveness and resistance to chemotherapy [56]. The successful initiation of programmed cell death by the active p53 protein is important for determining how tumor cells respond to different chemotherapy medications, with a lack of p53 potentially leading to heightened resistance to chemotherapy [26, 57]. p53 is a central transcription mediator associated with hundreds of target genes [58]. It is imperative to examine the relationship between noninvasive markers and immunohistochemical markers in order to elucidate the molecular mechanisms responsible for spatial heterogeneity. Furthermore, broadening their utility in areas such as prognosticating patient survival with neoadjuvant chemotherapy and assessing the effectiveness of neoadjuvant chemotherapy is essential.

There were specific constraints in this research. First, the small size of the cohort may have impacted the generalizability of the findings. Although PET/CT has excellent accuracy for identifying metastatic lesions, its high cost limits its use. However, with the continuous popularization of PET/CT, more and more patients are likely to have this imaging procedure. Second, owing to the lack of mature machine technology for segmentation, we used manual segmentation for VOI delineation, which could lead to inconsistencies. Third, the digital whole slide images of the primary lesion were used to calculate the immunohistochemical score. Due to the presence of intratumoral heterogeneity within the tumor, the immunohistochemical score may differ slightly from that of the whole tumor. However, the accuracy and stability were still improved compared to conventional manual scoring methods using artificial intelligence algorithms. Fourth, it is crucial to recognize that our findings were only relevant to individuals with HGSOC who have at least one metastatic tumor.

## 5 Conclusions

Models consisting of quantitative ^18^F‐FDG PET/CT spatial heterogeneity indicators based on solid tumor components can improve the predictive performance of platinum resistance in HGSOC. There was a correlation between heterogeneity indicators and the sores of Ki‐67 and p53, which may verify the feasibility of using spatial heterogeneity indicators in clinical practice at the molecular level. Future research aims to create a standardized integrated software that combines data from multiple dimensions to automatically compute spatial heterogeneity metrics, assess risk levels, predict potential prognosis, and recommend personalized treatment strategies.

## Author Contributions

**Xin Zhang:** investigation (equal), visualization (equal), writing – original draft (lead). **Yuhe Lin:** investigation (lead), methodology (equal). **Dianning He:** methodology (equal), software (lead), visualization (equal). **Mingli Sun:** data curation (equal), resources (equal). **Lanlan Xu:** investigation (equal). **Zhihui Chang:** writing – review and editing (equal). **Zhaoyu Liu:** conceptualization (equal), funding acquisition (lead), resources (equal). **Beibei Li:** formal analysis (lead), supervision (lead).

## Ethics Statement

Ethics Committee of Shengjing Hospital of China Medical University approved this study (2021PS881K) in order to uphold ethical standards and safeguard the rights and interests of the study participants. Because the study was conducted retrospectively, the Ethics Committee has granted permission to waive the requirement for written informed consent.

## Conflicts of Interest

The authors declare no conflicts of interest.

## Supporting information


Appendix S1.

## Data Availability

The datasets that underpin the conclusions drawn in this article are accessible on GitHub at the following link: https://github.com/beibei22008/platinum‐resistance_HGSOC.
