# Supplementary material for: 18F‐Fluoro‐2‐Deoxyglucose Positron Emission Tomography/Computed Tomography Measures of Spatial Heterogeneity for Predicting Platinum Resistance of High‐Grade Serous Ovarian Cancer
Source: Cancer Med. 2024 Oct 22;13(20):e70287. doi: 10.1002/cam4.70287 (PMC11494247; doi:10.1002/cam4.70287)
Supplement: Supplementary file 1 — Appendix S1. [file CAM4-13-e70287-s001.docx]

**Appendix S1.1**


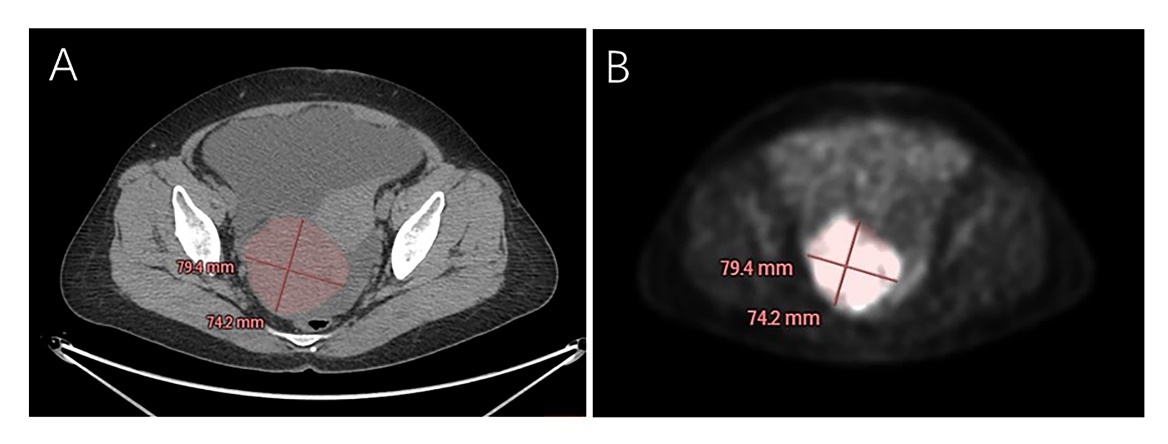


Figure S1.1: Volume of interest delineation of CT (A) and PET (B) images

**Appendix S1.2 Abdominopelvic regions assessment**

**
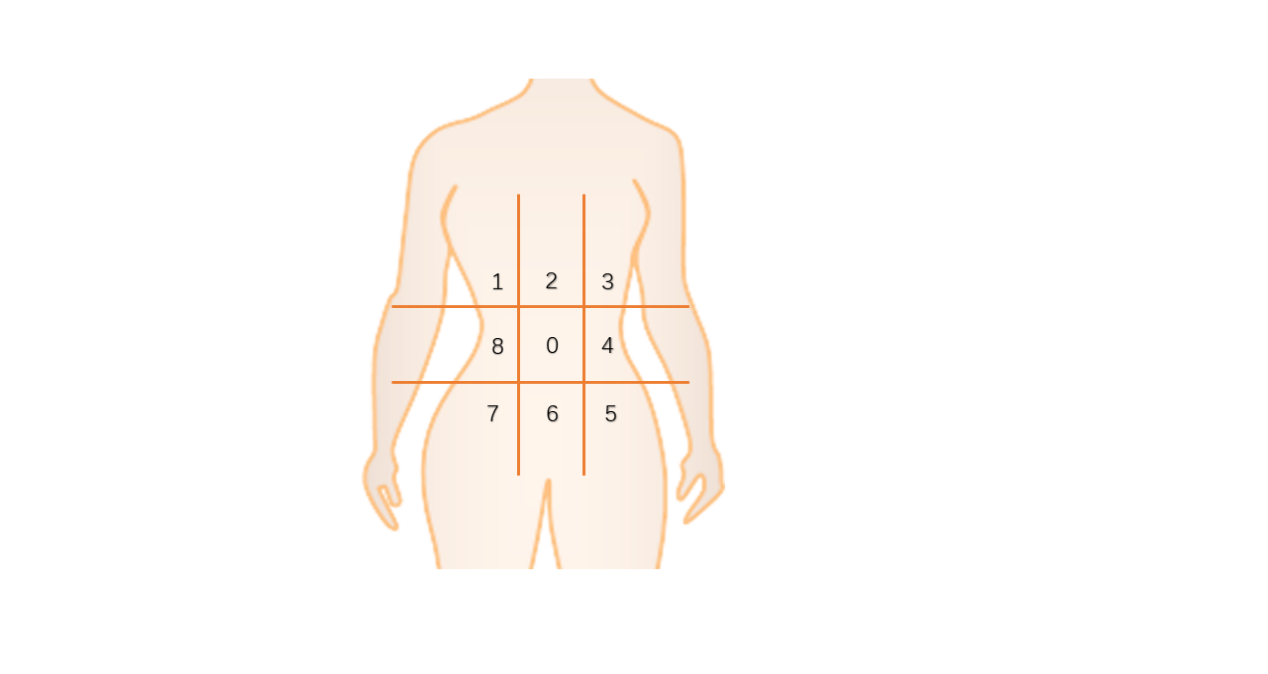
**

Figure S1.2: Abdominopelvic regions (AR0-AR8).

Table S1.2. Anatomical structures included in each region ^1-3^

| Regions | Location | Anatomic structures included |
| --- | --- | --- |
| AR-5 | left lower | the pelvic side wall lateral to the sigmoid colon and the sigmoid colon itself |
| AR-6 | pelvis | the female internal genitalia with ovaries, tubes, and uterus; bladder; cul-de-sac of Douglas; and the rectosigmoid colon |
| AR-7 | right lower | the right pelvic side wall and the base of the cecum, including the appendix |
| AR-4 | left flank | the descending colon and the left abdominal gutter |
| AR-0 | central | midline abdominal incision, the greater omentum and the transverse colon |
| AR-8 | right flank | the right abdominal gutter and the ascending colon |
| AR-3 | left upper | the undersurface of the left hemidiaphragm, the spleen, the tail of the pancreas, and the anterior and posterior surfaces of the stomach |
| AR-2 | epigastrium | the epigastric fat pad, the left lobe of the liver, the lesser omentum, and the falciform ligament |
| AR-1 | right upper | superior surface of the right lobe of the liver and the undersurface of the right hemidiaphragm |

Abbreviations: AR: area.

**Appendix S1.3**

Initially, we assessed the computed tomography (CT) images of each volume of interest (VOI) to derive four conventional CT measurements: pixel number or volume (Vol), CT value in Hounsfield units (HU), major axis length (Maj), and minor axis length (Min). Subsequently, we analyzed the positron emission tomography (PET) images of each VOI to determine the pathological area and obtain four conventional PET measurements: maximum standardized uptake value (Max), mean standardized uptake value (Mean), peak standardized uptake value (Peak), and total lesion glycolysis (TLG) within the pathological area. Each patient contained 2-10 VOIs, and all conventional measurements of these VOIs underwent secondary statistical analysis, which included the following 13 statistical measures: (1) central tendency statistics, comprising the mean, median, mode, and quartile deviation (R); (2) discrete trend statistics, including standard deviation (Std_dev), standard error of the sample mean (SM), variance, range, coefficient of variation (CV), corrected sum of squares (CSS), and uncorrected sum of squares (USS); and (3) distribution statistics, specifically kurtosis and skewness. Using the conventional measurement Max as an example, a total of 13 statistical indicators were derived following secondary statistical analysis. These indicators included Max_Mean, Max_Median, Max_Mode, Max_td_dev, Max_Variance, Max_Range, Max_CV, Max_CSS, Max_USS, Max_R, Max_SM, Max_Skewness, and Max_Kurtosis. Given that there were 8 conventional measurements (4 from CT and 4 from PET), each patient was assigned 13 × 8 = 104 spatial heterogeneity indicators at the patient level, as detailed in Table S1.3.

Table S1.3 Spatial heterogeneity indicators generated conventional features

| Conventional features | Spatial heterogeneity indicators generated |
| --- | --- |
| pixel number or volume (Vol) | Vol_Mean Vol_Median Vol_Mode Vol_Std_dev Vol_Variance Vol_Range Vol_CV Vol_CSS Vol_USS Vol_R1 Vol_SM Vol_Skewness Vol_Kurtosis |
| CT value (HU) | HU_Mean HU_Median HU_Mode HU_Std_dev HU_Variance HU_Range HU_CV HU_CSS HU_USS HU_R1 HU_SM HU_Skewness HU_Kurtosis |
| major axis length (Maj) | Maj_Mean Maj_Median Maj_Mode Maj_Std_dev Maj_Variance Maj_Range Maj_CV Maj_CSS Maj_USS Maj_R1 Maj_SM Maj_Skewness Maj_Kurtosis |
| minor axis length (Min) | Min_Mean Min_Median Min_Mode Min_Std_dev Min_Variance Min_Range Min_CV Min_CSS Min_USS Min_R1 Min_SM Min_Skewness Min_Kurtosis |
| maximum standardized uptake value (Max) | Max_Mean Max_Median Max_Mode Max_Std_dev Max_Variance Max_Range Max_CV Max_CSS Max_USS Max_R1 Max_SM Max_Skewness Max_Kurtosis |
| mean standardized uptake value (Mean) | Mean_Mean Mean_Median Mean_Mode Mean_td_dev Mean_Variance Mean_Range Mean_CV Mean_CSS Mean_USS Mean_R1 Mean_SM Mean_Skewness Mean_Kurtosis |
| peak standardized uptake value (Peak) | Peak_Mean Peak_Median Peak_Mode Peak_Std_dev Peak_Variance Peak_Range Peak_CV Peak_CSS Peak_USS Peak_R1 Peak_SM Peak_Skewness Peak_Kurtosis |
| total lesion glycolysis (TLG) | TLG_Mean TLG_Median TLG_Mode TLG_Std_dev TLG_Variance TLG_Range TLG_CV TLG_CSS TLG_USS TLG_R1 TLG_SM TLG_Skewness TLG_Kurtosis |

**Appendix S1.4**

The conventional models utilized clinical characteristic data and conventional evaluation indicators from CT and PET images. The clinical characteristic data encompassed variables such as age, CA125 levels, ascites volume (Ascites_volume), ascites character (Ascites_character), and the status of residual tumors or surgical status (Sur_status). Conventional evaluation indicators for CT and PET scans included the presence of lymph node metastasis in the pelvic cavity (Pelvic_LN), middle abdomen (Mid_LN), upper abdomen (Upp_LN), and distant areas (Dis_LN). Additionally, the number of regions exhibiting peritoneal metastatic lesions (Num_AR), the presence of metastatic lesions across nine specific areas (AR0-AR8), the invasion mode or pattern of peritoneal metastatic invasion (Pattern_of_invasion), the ratio of solid components (Solid_ratio), the volume or pixel number of the primary lesion (Vol), CT value (HU), major axis length (Maj), minor axis length (Min), maximum standardized uptake value (Max), mean standardized uptake value (Mean), peak standardized uptake value (Peak), and total lesion glycolysis (TLG) in the pathological area were also considered. The spatial heterogeneity indicators derived from texture features encompassed cluster site entropy (cSE), cluster diversity (cluDev), and cluster standard deviation (cludiss). The heterogeneity models incorporated spatial heterogeneity indicators based on both conventional and texture features. All the aforementioned data were utilized to develop the integrated models, as shown in Table S1.4.

Table 1.4 The collected data used to establish platinum resistance models

| Prediction models | Data |
| --- | --- |
| Conventional model | Age, CA125, FIGO_stage, Ascites_volume, Ascites_character, Sur_status, Pelvic_LN, Mid_LN, Upp_LN, Dis_LN, Num_AR, AR5, AR6, AR7, AR4, AR0, AR8, AR3, AR2, AR1, Pattern_of_invasion, Solid_ratio, Vol, HU, Maj, Min, Max, Mean, Peak, TLG (n=30) |
| Heterogeneity model | spatial heterogeneity indicators based on conventional features (n=104) and texture features (n=3) |
| Integrated model | All the data above (n=137) |

1. Lin CN, Huang WS, Huang TH, et al. Adding Value of MRI over CT in Predicting Peritoneal Cancer Index and Completeness of Cytoreduction. *Diagnostics (Basel)* 2021; 11 2021/05/01. DOI: 10.3390/diagnostics11040674.

2. Jacquet P and Sugarbaker PH. Clinical research methodologies in diagnosis and staging of patients with peritoneal carcinomatosis. *Cancer Treat Res* 1996; 82: 359-374. 1996/01/01. DOI: 10.1007/978-1-4613-1247-5_23.

3. Diaz-Gil D, Fintelmann FJ, Molaei S, et al. Prediction of 5-year survival in advanced-stage ovarian cancer patients based on computed tomography peritoneal carcinomatosis index. *Abdominal Radiology* 2016; 41: 2196-2202. DOI: 10.1007/s00261-016-0817-5.
